# Long-term behavioral adaptation of Oldowan toolmakers to resource-constrained environments at 2.3 Ma in the Lower Omo Valley (Ethiopia)

**DOI:** 10.1038/s41598-023-40793-3

**Published:** 2023-09-01

**Authors:** Anne Delagnes, Aline Galland, Brad Gravina, Pascal Bertran, Marion Corbé, Michel Brenet, Haregwin Belete Hailu, Fikeru Mekonenn Sissay, Bisrat Gebreegziabher Araya, Misganaw Gebremichael Woldetsadik, Jean-Renaud Boisserie

**Affiliations:** 1 PACEA, University of Bordeaux-CNRS, Pessac, France; 2 Musée National de Préhistoire, Les Eyzies, France; 3 Inrap-NAOM, Bègles, France; 4 PALEVOPRIM, CNRS-University of Poitiers, Poitiers, France; 5 Sapienza Università Di Roma, Rome, Italy; 6 University of Evora, Evora, Portugal; 7 Ethiopian Heritage Authority (EHA), Addis Ababa, Ethiopia; 8 Centre Français des Études Éthiopiennes, CNRS-Ministry of Europe and Foreign Affairs, Addis Ababa, Ethiopia

**Keywords:** Evolution, Environmental social sciences

## Abstract

The long stratigraphic sequence of the Shungura Formation in the Lower Omo Valley documents 3 million years (Ma) of hominin evolution, which, when combined with detailed paleo-depositional environmental data, opens new perspectives for understanding the complex interactions between hominin landscape use and the development of stone tool-mediated activities. Stone tool assemblages produced by *Paranthropus aethiopicus* and/or a species of early *Homo* from ~ 2.3 Ma*,* reflect their ability to deal with the raw material scarce environment of the Lower Omo Valley. It remains to be seen whether this activity can be related to a single, brief occupation event or the expression of an emergent new adaptation. Here we report on the newly investigated site complex of OMO 79, which produced the first evidence for multiple phases of hominin tool-making and use in the Shungura Formation. The development of this long-lasting techno-economic behavior marks a cognitive tipping point around 2.3 Ma in the Lower Omo Valley, evidenced by the adaptability of the early hominins to resource-constrained environments.

## Introduction

The earliest evidence for stone tool production in the Lower Omo Valley (southern Ethiopia) comes from Member F of the Shungura Formation, dated to between 2.324 ± 0.020 Ma and 2.271 ± 0.041 million-years (Ma)^1,2^, in an area that was frequented by hominins for more than 3 million years^3–5^. This emergent behavior is documented by a large number of hominin occupations in varied depositional settings, all related to the meandering river floodplain deposits of the ancestral Omo River. The sudden development of tool-assisted behaviors in Member F^6^ is unlikely linked to either biological or environmental factors as the two hominin taxa documented in Member F, *Paranthropus aethiopicus* and *Homo sp.*, are equally present in underlying Member E^4,5^ and from a similar environmental setting. Member F deposits are composed of low-energy alluvial sediments laid down by the meandering Omo River under semi-arid environmental conditions that emerged much earlier, as early as 2.8 Ma^7–9^. Importantly and in stark contrast with all other Early Pleistocene sites in eastern Africa^10–15^, the landscapes occupied by the Shungura hominins were devoid of predictable sources of abundant raw materials in the form of cobble conglomerates^6,16^. Whether this unprecedented behavioral adaptation of hominins to a raw material scarce environment is related to a unique, brief occupation or the expression of an emergent long-term tradition remains to be seen. Here we report on a recently discovered site complex, OMO 79, which provides important new data for the flexibility of early Oldowan toolmakers.

## Results

### Overview of the archeological occurrences in the Shungura Formation

**Figure 1:**
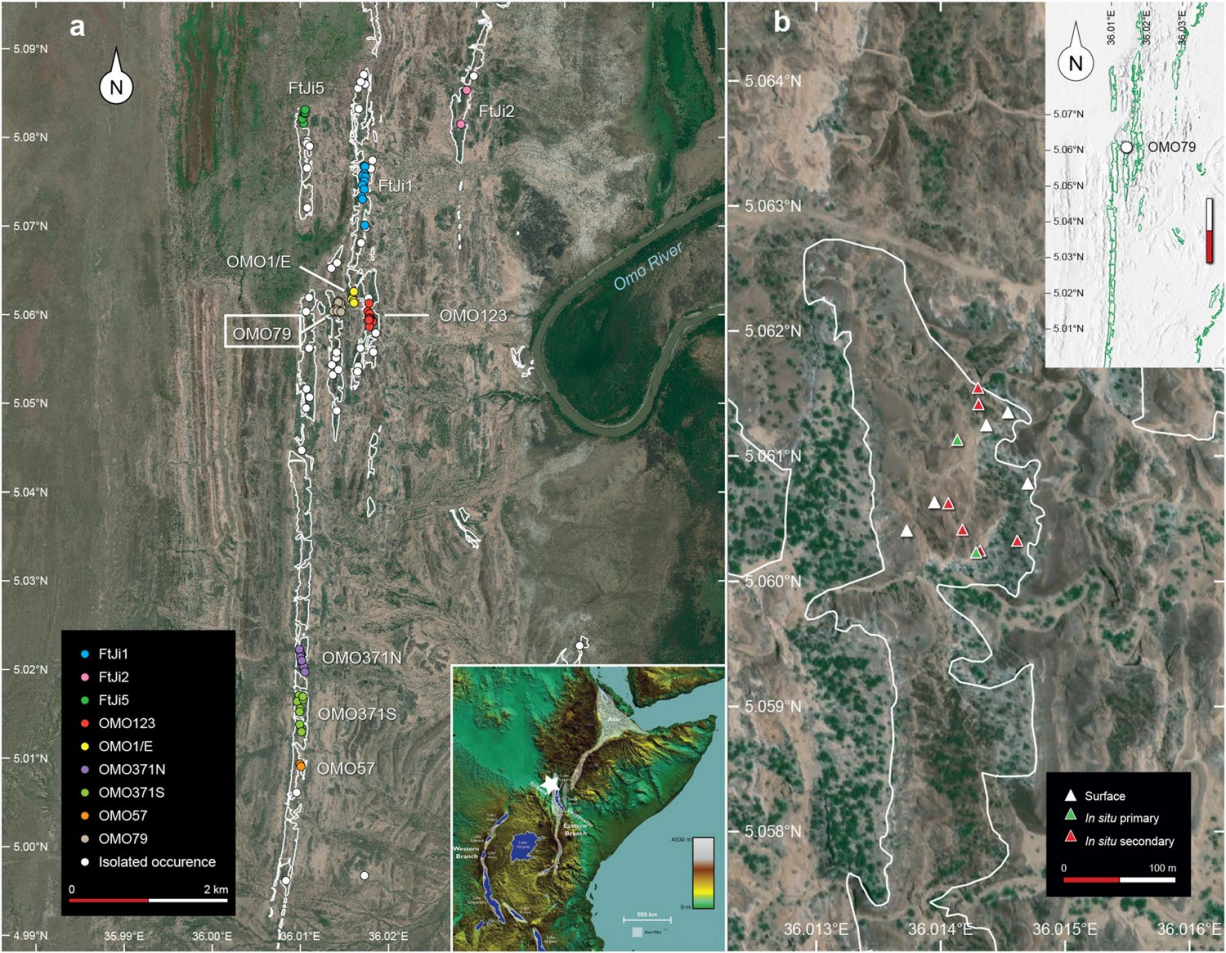
Satellite views of the Shungura Formation – Member F. (**a**) Archaeological sites complexes and occurrences in the northern outcrops of the Shungura Formation and in the broader context of the Eastern African Rift System. (**b**) Distribution and archaeological context of occurrences at OMO 79 and the site complex's position within the overall extension of Member F (green outline). Satellite images were obtained from Bing Aerial Maps and transformed using QGIS 3.28 LTR (https://www.qgis.org/en/site/about/index.html).

Extensive archaeological surveys and excavations in the Shungura Formation during the late 1960s and early 1970s^4^, followed by new field work since 2008^17,18^, confidently place the first appearance of stone tool manufacture in the lower part of Member F, around 2.3 Ma. Abundant paleontological collections, including hominin fossils, provide a wide range of data for exploring the interactions between environmental fluctuations and faunal communities from Member A, dated to ~ 3.6 Ma^18–20^. The more than one million years that separates these two members sees the appearance of the earliest known stone artifacts^21^ and Oldowan assemblages^10,22^ in Africa. In the Lower Omo Valley, no in situ stone tools have been recovered from sediments deposited during this time range despite considerable survey work and test trenches. In Member F, 56 in situ archaeological occurrences have been recorded, completed by 65 surface artifact concentrations found in close spatial and stratigraphic association (for more details see *SI Appendix*). Of these, 78 are distributed across nine different site complexes^17,18,23^ (Fig. 1a), which correspond to concentrations of at least two spatially distinct occurrences that can be directly connected stratigraphically. While major tuff deposits can be traced across the Shungura Formation, intercalated fluvial deposits preclude similar long-distance stratigraphic correlations, mainly due to the significant lateral variations induced by the migration of the ancestral Omo River across the floodplain, the discontinuous nature of outcrops and benchmark levels, as well as faults. The relative chronology of hominin occupations can be reliably built only through stratigraphic correlations observable at each site complex.

All nine site complexes of Member F (Fig. 1a) primarily record a unique occupation phase within a single layer of fine sediments. These occupations take the form of several primary or sub-primary occurrences (e.g. OMO 371N and OMO 371S), preserved in their original depositional layer, or multiple occurrences of artifacts remobilized in the same channel lag deposit, as at FtJi1^4,6,16,24^ and OMO 57^4,25^. A similar scenario of a unique occupation phase likely applies to site complexes with two artifact-bearing deposits: an upper archeological layer most often found in primary/sub-primary fine sediments from river bank deposits and a spatially offset lower channel lag containing redeposited artifacts from the upper layer which was eroded as the channel migrated^26^, as seen at FtJi2^4,6,24^, OMO 123^4,25^ and OMO 1/E. By contrast, OMO 79 documents 13 archaeological occurrences superimposed in several points of the site complex, which is consistent with artifacts deriving from multiple occupation phases, marked by distinct episodes of stone tool production, a unique situation in the Shungura Formation. The OMO 79 occurrences are spread over approximately 8000 m^2^ (Fig. 1b) in the lower part of Member F, in various sedimentary units that can be tied to discrete river margin settings. These occurrences correspond to well-defined concentrations of stone tools recovered on the slopes, or top and on the slopes of sedimentary hills, excluding any remains scattered along the base^27^. In situ material was found in 4 occurrences while the others are surface concentrations. The original layer of these surface occurrences could be reliably identified based on the position of the uppermost pieces, the limited vertical dispersion of the artifacts, their clear spatial association with the same sedimentological layer that can be followed across the entire site complex and sediment adhering to the artifacts. The integrity of the assemblages is evidenced by their marked technological homogeneity and the total absence of surface remains from more recent periods in this sector of the Shungura Formation.

### Stratigraphy and depositional environments at OMO 79

**Figure 2:**
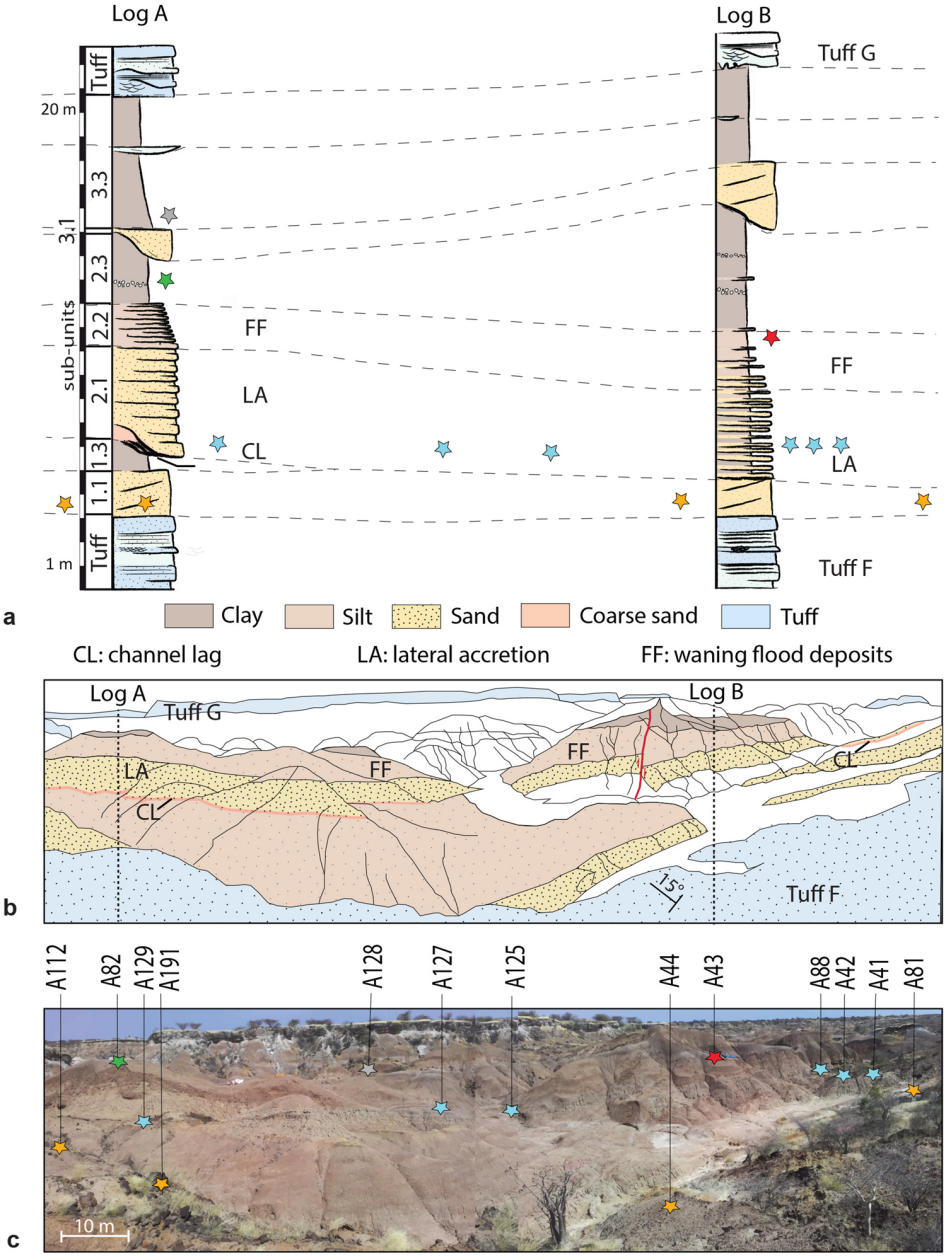
Stratigraphy and distribution of OMO 79 occurrences. (**a**) Stratigraphic logs at A82 (log A) and A43 (log B). (**b**) Schematic panoramic reconstruction of the OMO 79 sequence, seen from the eastern edge of the OMO 79 deposits. (**c**). Distribution of archaeological occurrences seen from the same position (phase 1: orange stars, phase 2: blue stars, phase 3: red star, phase 4: green star, phase 5 (?): grey star).

**Figure 3:**
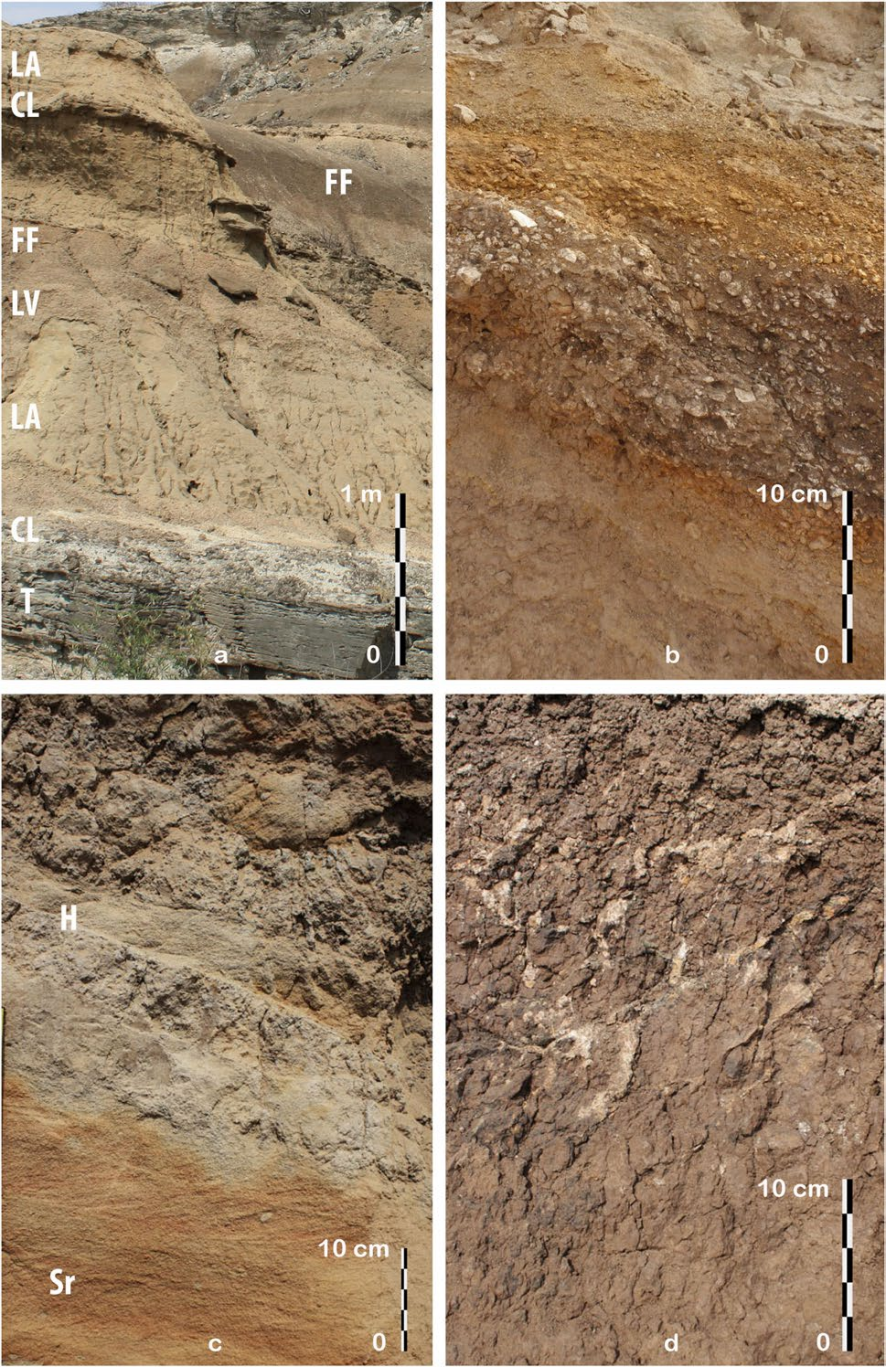
Views of the main lithofacies. (**a**) General view of the OMO 79 deposits (occupation phases 1 and 2), T: volcanic tuff, CL: channel lag, LA: lateral accretion unit (point bar), LV: heterolithic levee/upper point bar package, FF: floodplain fines. (**b**) Channel lag composed of CaCO3 concretions, quartz gravel, rip-up clasts, and an in situ quartz artifact in secondary context (occurrence A127). (**c**) Upper point bar ripple cross-laminated sands (Sr) and heterolithic sediment package (H). (**d**) Clayey silt floodplain deposit with CaCO_3_ concretions.

The 70 m-thick sedimentary deposits of OMO 79 are tilted 15° to the west and comprise four superimposed upward-fining fluvial units between Tuff F and Tuff G (Figs. 2 and 3a).

The main lithofacies are as follows:

- channel lag deposits (CL): massive, trough cross-bedded, decimeter-thick sandy gravel units with an erosional base, that primarily comprise pedogenic concretions and rip-up clasts, as well as small (1–2 cm) gravels of volcanic and metamorphic rocks (Fig. 3b);
- point bar deposits: large-scale, low-angle strata including planar and trough cross-bedded sands displaying a paleocurrent perpendicular to the large-scale stratification (lateral accretion units, LA) (Fig. 3a,c)—the absence of major erosional surfaces within the lateral accretion packages is consistent with each sand body corresponding to a single-storey point bar;
- levee and upper point bar deposits (LV): heterolithic sediment package comprised of interbedded deposits of sand and silt (Fig. 3c);
- fine-grained floodplain deposits (FF): decimeter-thick, massive silty clay and clay beds with a prismatic structure occasionally interrupted by silt or sand layers, in which pedogenic carbonate nodules form recurrent horizons (Fig. 3d).

The overall sequence records floodplain accretion and lateral migration of the paleo-Omo channel across the floodplain, in a context of the creation of accommodation space following subsidence and lake transgression. The reconstruction of depositional environments, sedimentary processes and stratigraphic architecture provide evidence for at least four hominin occupation phases above Tuff F, each composed of either a single or multiple archaeological occurrences (Fig. 2 and Table 1).

**Table 1:** OMO 79 archaeological occurrences.

Occurrence	Nb surf. artifacts	Nb in situ artifacts	Total Nb	Lithofacies	Sedimentary sub-unit	Occupation phase
OMO A44	91	0	**91**	Gm	1.1	1
OMO A81	150	0	**150**	Gm	1.1	1
OMO A112	129	0	**129**	Gm	1.1	1
OMO A191	53	0	**53**	Gm	1.1	1
OMO A41	0	1	**1**	Gm	2.1	2
OMO A42	165	0	**165**	Gm	2.1	2
OMO A88	55	0	**55**	Gm	2.1	2
OMO A125	37	0	**37**	Gm	2.1	2
OMO A127	11	1	**12**	Gm	2.1	2
OMO A129	201	0	**201**	Gm	2.1	2
OMO A43	127	121	**248**	Sh & Fl	2.2	3
OMO A82	318	2	**320**	Fm	2.3	4
OMO A128	11	0	**11**	Fm	3.3?	5?

Number of surface *vs *in situ artifacts collected at OMO 79; lithofacies (Gm: massive gravels, Sh: horizontally bedded sands, Fl: laminated fines, Fm: massive fines), sedimentary sub-units and corresponding occupation phases.

#### First phase of hominin occupation

**Figure 4:**
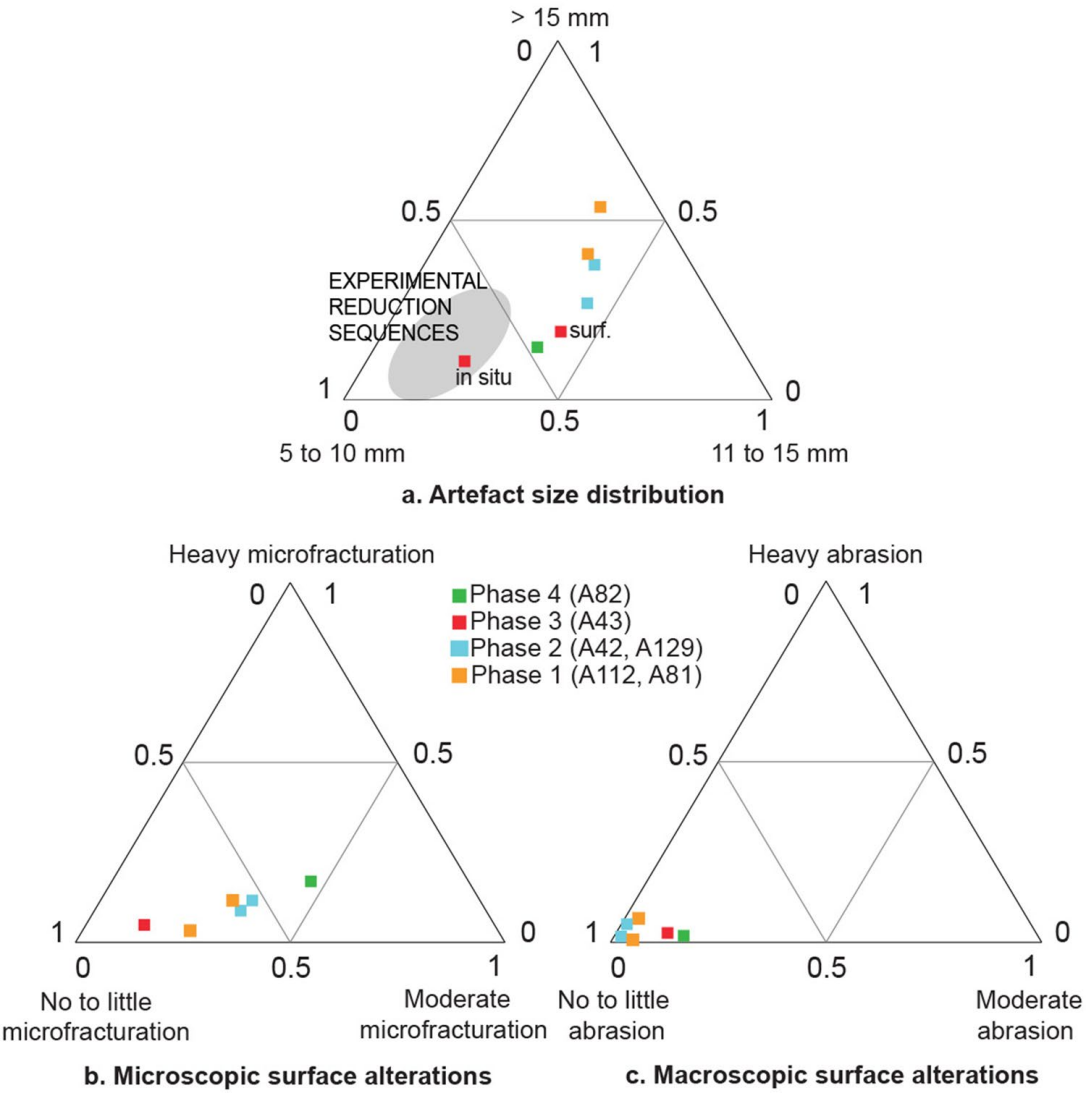
Size distribution and surface alterations of the OMO 79 lithic assemblages. (**a**) Artifact size distribution of the main lithic assemblages compared with experimental reduction sequences. (**b**) Microscopic surface alterations of the crystals and ridges on quartz artifacts. (**c**) Macroscopic surface alterations (details concerning macro- and micro-alterations are provided in Fig. 5). The experimental data used in (**a**) come from an experimental collection produced by five knappers, four novices and one expert knapper, who reduced a total of 37 quartz pebbles, resulting in 1598 products > 1 cm using a single technique, either bipolar or free-hand, and for the sole purpose of obtaining as many sharp-edged products as possible, until the core was exhausted. Quartz pebbles used for the experiments were collected in the Lower Omo Valley, and selected to be large enough for producing flakes, i.e., > 5 cm in maximum length, regardless their knapping qualities. The experimental dataset is available on Nakala (https://doi.org/10.34847/nkl.3e292r29) and detailed in^26,28^.

**Figure 5:**
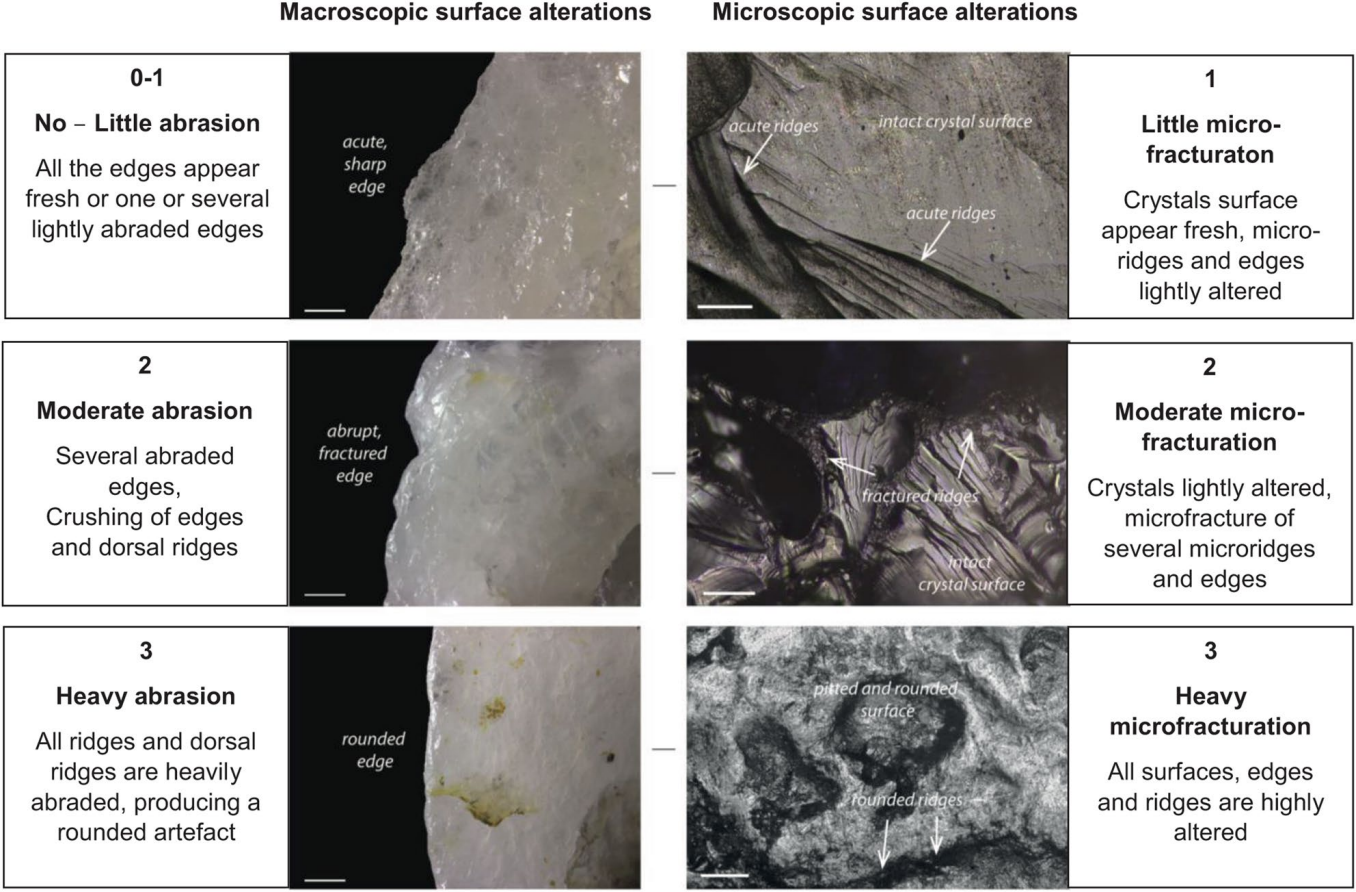
Classes of altiration on quartz artefacts. Description of the alteration stages at macroscopic and microscopic scales (macroscopic scale is 1 mm; microscopic scale is 100 μm).

Four archaeological occurrences (A44, A81, A112, A191) were found scattered over the 150 m long OMO 79 outcrop, in the basal part of a sand body overlying Tuff F (sub-unit 1.1) (Fig. 2 and Table 1). These occurrences represent remnants of occupations redistributed in a channel following bank erosion, and ultimately redeposited downstream as small secondary concentrations mixed with lag gravels. Artifact size distributions for the two largest assemblages, A81 and A112, point to the heavy winnowing of the fine fraction (Fig. 4a). Macro- and microscopic analysis of surface alterations of quartz artifacts (Fig. 5) produced contrasting results. More than 90% of artifacts exhibit little to no macroscopic abrasion (Supplementary Fig. S1), whereas moderate and heavy microfracturation (Supplementary Fig. S1) account for 43% and 32% of the analyzed sample from A112 and A81, respectively (Fig. 4b,c).

#### Second phase of hominin occupation

Six archaeological occurrences (A41, A42, A88, A125, A127, A129) were found in the sand layer (sub-unit 2.1) overlying the deposits from phase 1 (Fig. 2 and Table 1). These occurrences equally correspond to small secondary concentrations associated with gravel lags (Figs. 2 and 3b). The distance of transport and, hence, the original depositional context cannot be precisely determined. Isolated in situ artifacts were found at A41 and A127 (Fig. 3b). Artifact size distribution for the largest assemblages, A42 and A129, are readily comparable with those of phase 1 (Fig. 4a) and thus reflect similar water transport and size sorting. Identical to phase 1, more than 90% of the quartz artifacts bear little to no macroscopic abrasion (Supplementary Fig. S1), while 47% and 43% of artifacts from A42 and A129, respectively, exhibit moderate to heavy microfracturation (Fig. 4b and Supplementary Fig. S1). Among the abundant faunal remains collected during surface surveys at A42, abraded bones account for 9.14% (n = 1829) of the total assemblage . No clear traces of anthropic surface modifications were identified^26^.

#### Third phase of hominin occupation

This phase is represented by a single, primary occurrence, A43, within a 20 to 30 cm thick heterolithic unit at the top of a point bar sequence (sub-unit 2.2) (Fig. 2) that yielded both in situ and surface artifacts (Table 1 and Supplementary Fig. S2). In this overbank deposit context, water transport of archeological material is likely to have been limited. Accordingly, artifact size distributions fall within, or close to the zone of experimental reduction sequences (Fig. 4a). The majority of A43 surface and in situ artifacts show little to no macroscopic abrasion (91%: Supplementary Fig. S1), while moderate to heavy microfracturation is present on 24% of artifacts (Fig. 4b and Supplementary Fig. S1). The presence of a small proportion of heavily altered pieces (2% and 7% at macro- and microscopic scale, respectively) suggests they were transported over significant distances before being mixed with fresh pieces.

#### Fourth phase of hominin occupation

A single occurrence, A82, located at the top of two adjacent hills composed of fine-grained floodplain deposits (sub-unit 2.3) (Fig. 2), provides evidence for a fourth occupation phase. Only the southernmost hill (A82 South) yielded two in situ artifacts, 30 cm below the ground surface in a clayey layer with slickensides. This layer has been entirely eroded from the second hill (A82), which did not yield any in situ material, whereas surface artifacts were abundant on its slopes (Supplementary Fig. S3). Artifact-size distribution (Fig. 4a) and macroscopic surface alterations (88% of artifacts bear little to no macroscopic abrasion: Supplementary Fig. S1) suggest the assemblage to have experienced very little winnowing and only limited water transport. By contrast, A82 exhibits the highest proportion of moderate to heavily microfractured artifacts (63%: Supplementary Fig. S1) among all phases (Fig. 4b).

#### Fifth phase of hominin occupation (?)

The uppermost A128 occurrence can potentially be tied to a fifth phase of hominin occupation on the floodplain, as evidenced by a small concentration of artifacts atop a sedimentary hill capped by clay and carbonated nodules (unit 3.3), some 9 m above A82 (Fig. 2). Two trenches cut into a thick and compact clayey deposit did not provide any in situ material, making it likely that the artifact-bearing deposit has been completely eroded.

### Assemblage taphonomy and composition

The taphonomic analysis of the OMO 79 lithic assemblages reveals contrasting patterns of artifact preservation. Assemblages from primary and secondary contexts share similarly high proportions of quartz artifacts bearing little to no macroscopic surface alterations (Fig. 4c). Macroscopic abrasion due to water transport^29,30^ is limited for all depositional contexts. On the other hand, the small proportion of heavily abraded quartz fragments in all assemblages reflects the introduction of objects with a different depositional history. The impact of water transport is more apparent in the artifact size distributions, with phases 1 and 2 characterized by heavily size-sorted assemblages. By contrast, size sorting is absent or limited in the assemblages of phases 3 and 4, both in primary or sub-primary depositional contexts. A comparison of micro- and macroscopic surface alterations (Fig. 4b,c) provides complementary information. Both size-sorted (i.e., water-transported) and unsorted (non-transported) assemblages contain moderately to heavily microfractured artifacts (Fig. 4b), the highest amounts being found in the clay floodplain deposits of A82. Microscopic alterations are thus most likely connected to processes other than water transport, namely the vertical displacement of artifacts by argiliturbation^31,32^, as evidenced by the presence of numerous slickenslides at A82.

**Figure 6:**
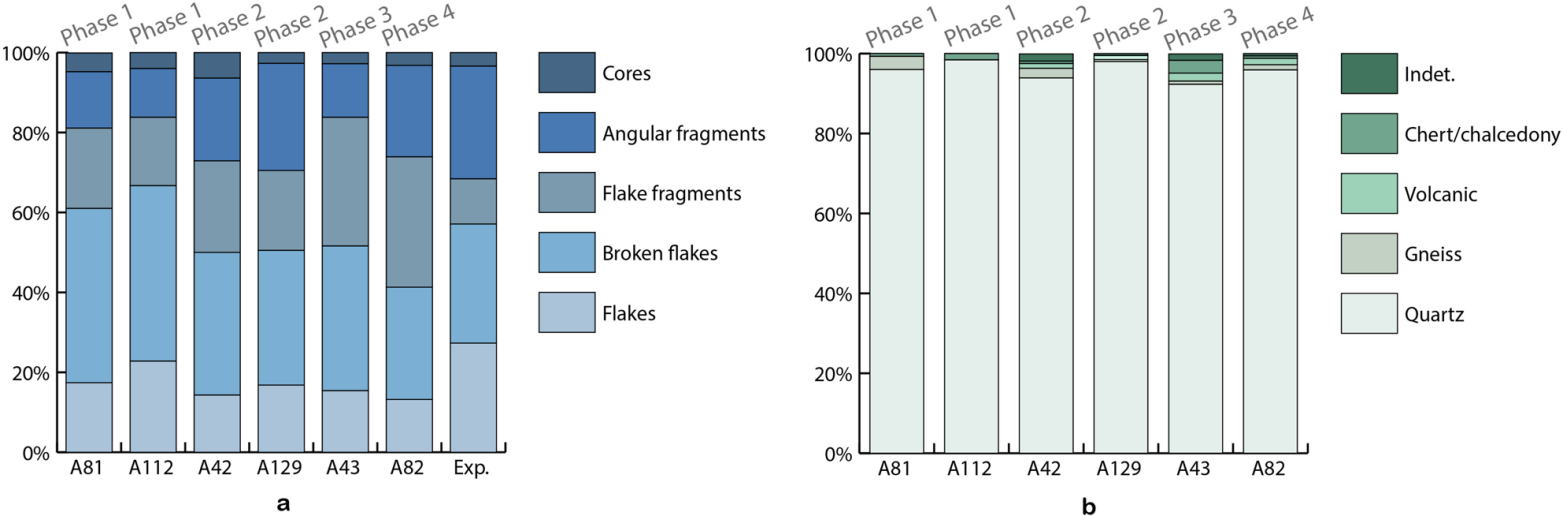
Main attributes of the OMO 79 lithic assemblages. (**a**) Technological composition of the archaeological assemblages compared with experimental data (Exp.). (**b**) Raw material frequencies. Occurrences A81, A42, A43, A82, A112, A129, with a total number of artifacts > 100), including –all lithics > 1 cm, excluding the unmodified clasts.—see Supplementary Table S1 for data source and https://doi.org/10.34847/nkl.3e292r29 for the experimental dataset.

**Figure 7:**
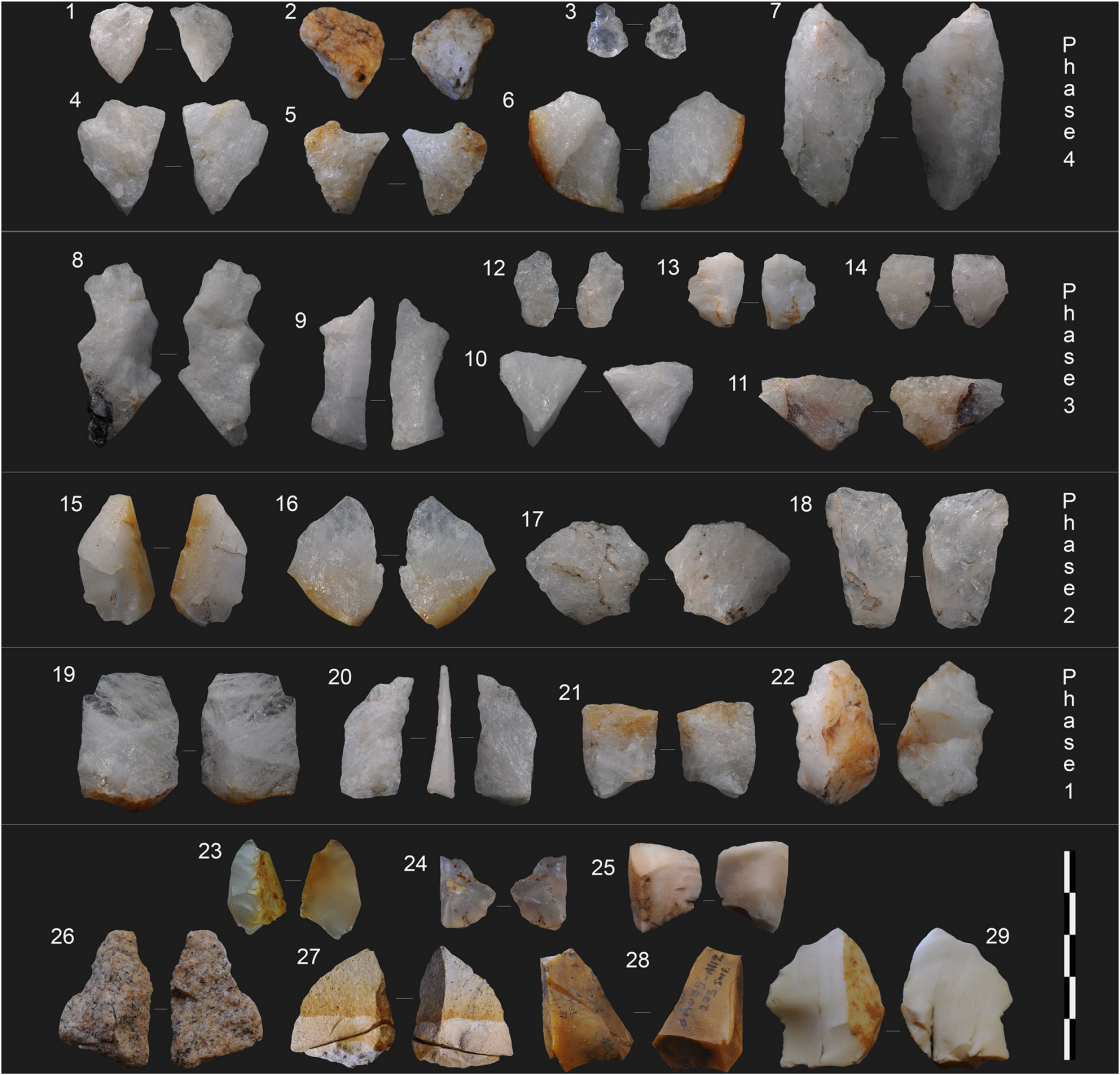
Lithic artifacts from OMO 79. 1 to 7: quartz flakes and broken flakes from A82; 8 to 14: quartz flakes and broken flakes from A43; 15 to 18: quartz flakes and broken flakes from A42 (15–16) and A129 (17–18); 19 to 22: quartz flakes and broken flakes from A112; 23–24: chalcedony flakes from A43; 25, 28–29: chert flakes and broken flakes from A129, A112 and A82, respectively; 26: gneiss flake from A43; 27: lava flake with a refitted proximal fragment from A43. Picture and photo montage by AD, with Adobe Photoshop 22.5.

The OMO 79 lithic assemblages (Figs. 6, 7 and Supplementary Table S1) share features that are common to all Member F assemblages^16,17,24,25,33,34^. They result from a simple core-flake technology using both free-hand and bipolar percussion of small, primarily quartz river pebbles, for the production of cutting-edged flakes. The significance of the bipolar technique varies from one assemblage to another. The proportion of diagnostic bipolar flakes indicates a predominant, if not exclusive, use of this percussion technique in the A43 assemblage and a more balanced use of free-hand and bipolar techniques in the A82 assemblage, with 11.7% and 3.4% of diagnostic bipolar flakes, respectively^28^. The length, width and thickness of flakes and flake fragments do not vary significantly from one assemblage to another, with mean maximum length values just above 2 cm and the presence in all assemblages of a few products greater than 4 cm (Supplementary Fig. S4). Split fractures are frequent, which results in a diversity of flake fragments^33^, including products with a back formed by a lateral break opposite or adjacent a cutting edge, and a large proportion of angular fragments (Supplementary Table S1). Flakes appear to have been used unmodified. The homogeneity of the assemblages from Member F and their particular appearance compared with all other early Oldowan assemblages are linked to the size and mechanical constraints imposed by the local quartz pebbles rather than to distinct technological behaviors or skills^28^.

The strong predominance of quartz is the result of a selective raw material procurement^17^^.^ Raw materials other than quartz are represented by rare, isolated products from distinct pebbles. Locally available raw materials are scarce, difficult to access and of poor quality controlling reduction. The only place where pebbles greater than 2 cm have been found at OMO 79 is the gravel lag deposit of A112 (phase 1). An exhaustive survey of this area produced 79 pebbles, of which only five were of knappable size (i.e. greater than 5 cm in maximum length). Such a scarcity of exploitable raw materials suggests that the lag materials could not have served as a primary source of raw materials, in addition to the fact that their position as bed load in an active channel would render them difficult if not impossible to access. This pattern applies to the whole of the Shungura Formation (Member F). A significant part of the raw materials might thus have been brought from distant sources^6,16^, the closest one being the ephemeral channels of the Hamar Range alluvial fans, some 3 to 15 km to the east. Assemblage composition (Fig. 6 and Supplementary Table S1) shows both end-products (i.e., flakes and broken flakes with efficient cutting edges, angle < 60°), and by-products (angular fragments, cores) to be present in proportions consistent with core reduction being partially carried out on-site. These multiple tool-assisted activities are also consistent with relatively non-ephemeral hominin occupations.

## Discussion

The OMO 79 site complex provides the first example of multiple, well-defined hominin occupation phases in the Shungura Formation. Spread over a period of several thousand years in the lower part of Member F, the successive phases of hominin occupation were located on the margins of the paleo-Omo River. The archaeological occurrences of phases 3 and 4 (and likely 5) can be confidently placed on an upper point bar (phase 3: A43) and the proximal floodplain of the paleo-Omo River (A82: phase 4 and presumably A128: phase 5). The absence of archeological sites in the uppermost sedimentary units of Member F, which correspond to distal floodplain deposits, is congruent with a strong correlation between hominin settlement and river margin environments^26^. This echoes the recurrent correlation of hominin localities with riverine and lacustrine contexts^35^ or springs^36^ in the African Late Pliocene and Early Pleistocene record. River floodplains in semi-arid environments are characterized by heavily contrasting habitats, with an abrupt shift from gallery forests with dense perennial vegetation to edaphic grasslands dominated by annuals and forbs on the floodplain^37^. In this savannah grassland context, point bars would have been particularly attractive for hominins, as suggested by OMO 79 (A43 and A82), as well as Gona^38^ and Hadar^39^ in the Afar Depression. Distributed at regular intervals along the river, these highly productive patches of vegetation would have provided hominins with year-round predictable resources. Beyond an easy access to water, which also attracted other large predators, point bars likely afforded hominins with shelter, including large trees.

What is unique to OMO 79, as well as to the low energy fluvial deposits of the Lower Omo Valley in general, is the lack of abundant local raw materials suitable for stone tool manufacture^16,24^. This “resource-rich, stone-poor”^40^ environment contrasts with all other eastern African Early Pleistocene sites in contexts with a wide variety of locally available resources, including abundant and easily accessible raw materials in cobble conglomerates^10–14^. Regionally, several dozen kilometers south-east and south of the Shungura Formation, at Fejej and Nachukui, cobble conglomerates offered abundant raw materials to Oldowan groups for the manufacture of stone tools^14,41^.

The OMO 79 record reveals hominin behavioral adaptations to a raw material scarce environment not to result from a single, ephemeral phase of hominin settlement in the Lower Omo floodplain. Instead, hominins repeatedly occupied the area as part of multi-purpose, techno-economic activities, a behavior rooted in a long-lasting local tradition that emerged around 2.32 Ma in the lower part of Member F and continued until ca. 2.0 Ma, in the lower part of Member G, under relatively similar environmental conditions^42^. The flexibility of the Oldowan groups is well documented and supported, in particular, by the widespread distribution of early Oldowan sites across Africa from possibly as early as 2.9 Ma, as recently evidenced by new discoveries in eastern and northern Africa^43,44^. The adaptation of the Oldowan toolmakers to varied environments was fostered by the learned anticipation of raw materials properties and mechanical constraints, as evidenced for instance at Gona^12^ and Lokalalei 2C^14,45,46^.

The habit of regularly exploiting raw material poor environments is, however, exclusive to the Lower Omo Valley. Around 2.5 Ma in the Bouri Formation (Hata Member), in an environment similarly devoid of locally available raw materials, hominins only left behind isolated, scattered artifacts^47^, which strongly contrasts with the abundance and recurrence of archaeological occurrences in the Shungura Formation. This new adaptation of early Oldowan groups to the environment from 2.3 Ma onward echoes but predates discrete forms of complex responses to varied environments and related resources, as reported from ~ 2.0 Ma, at Kanjera South^48^, Olduvai Gorge^49^ and Koobi Fora^50^ in particular. While there is no doubt that this shift signals a tipping point in the Lower Omo Valley, more data are needed to test this assumption at the broader scale of the Eastern African Rift System. The Shungura Formation nevertheless adds an important new facet to the growing picture of the behavioral flexibility of early Oldowan groups.

## Methods

The OMO 79 site complex was discovered in 2008 during extensive archaeological surveys in Member F. It is situated some 400 m west of the OMO 123 site complex, where Jean Chavaillon carried out extensive field work between 1972 and 1976^25,51^. All of the OMO 79 archaeological occurrences have been extensively surveyed, inventoried, with surface and in situ material piece-plotted with a Total Station during the 2014 and 2018 field seasons. Test excavations and trenches were made at four occurrences (A128, A129, A82 and A43) following the identification of in situ or sub-surface artifacts and/or when abundant surface artifacts were observable (for more details, see Supplementary Informations*)*. Sediments from excavated occurrences were systematically dry sieved with a 3 mm mesh. At two occurrences where the faunal remains were particularly abundant (A42 and A112), all fauna and lithic artifacts were collected following the scraping and screening of the loose, upper 5 cm of sediments over ca. 20 m^2^ area.

The sedimentological study is based on data acquired during two field missions (by PB in 2014 and MC in 2018) and combines conventional sedimentological and stratigraphical observations of several laterally-logged sections in Member F, with lithofacies and stratigraphic architecture characterized via physical bed correlations. The analysis of artifact size distribution follows the method described by Bertran et al.^52^, and uses experimentally-derived size classes specifically adapted to bipolar technology using quartz pebbles collected in the Lower Omo Valley. All occurrences that yielded over 100 artifacts with a maximum dimension of more than 2 cm (n = 6) were included in the analysis of artifact surface alterations. In total, 398 artifacts from the Omo 79 site complex (Supplementary Fig. S1) were observed for macro- and microscopic surface alterations by AG. The recording of macroscopic alterations focused on the examination of edges and the main ridges of the artifacts while the recording of microscopic alterations considered the edges and crystal ridges on the widest surface of the artifacts (*see* Fig. 5 for the description of macro- and microscopic alteration stages).

### Supplementary Information

Supplementary Information.

## Data Availability

All data generated or analyzed during this study are included in this published article and its supplementary information file, or publicly accessible through Nakala data repository.
