# Supplementary material for: Long-term behavioral adaptation of Oldowan toolmakers to resource-constrained environments at 2.3 Ma in the Lower Omo Valley (Ethiopia)
Source: Sci Rep. 2023 Sep 1;13:14350. doi: 10.1038/s41598-023-40793-3 (PMC10474039; doi:10.1038/s41598-023-40793-3)
Supplement: Supplementary file 1 — Supplementary Information. [file 41598_2023_40793_MOESM1_ESM.pdf]

Supplementary Informations

Long-term behavioral adaptation of Oldowan toolmakers to resource-constrained environments at 2.3 Ma in the Lower Omo Valley (Ethiopia)

Anne Delagnes[1*], Aline Galland[1], Brad Gravina[2,1], Pascal Bertran[3,1], Marion Corbé[4], Michel Brenet[3,1+], Haregwin Belete Hailu[5+], Fikeru Mekonenn Sissay[6+], Bisrat Gebreegziabher Araya[1+], Misganaw Gebremichael Woldetsadik[1,7+], Jean-Renaud Boisserie[4,8,+]

[1] PACEA, University of Bordeaux-CNRS, Pessac, France
[2] Musée National de Préhistoire, Les Eyzies, France
[3] Inrap-NAOM, Bègles, France
[4] PALEVOPRIM, CNRS-University of Poitiers, Poitiers, France
[5] Sapienza Università Di Roma, Italy
[6] University of Evora, Portugal
[7] Ethiopian Heritage Authority (EHA), Addis Ababa, Ethiopia
[8] Centre Français des Études Éthiopiennes, CNRS-Ministry of Europe and foreign affairs, Addis Ababa, Ethiopia.

[*] Corresponding author - anne.delagnes@u-bordeaux.fr
[+] these authors contributed equally to this work

Overview on the archaeology in the Shungura Formation

The Shungura Formation is situated in the Lower Omo Valley of southwestern Ethiopia and, along with the Koobi Fora, Nachukui, Usno, Mursi Formations, forms part of the Plio-Pleistocene deposits of Turkana Depression defined as the Omo Group. Member F of the Shungura Formation outcrops over an area of approximately 3.3 km^2 and comprises nine main site complexes distributed along eroded, westward tilted deposits that are oriented north-south. Five site complexes were discovered in the late 1960s and 1970s as part of the International Omo Research Expedition – IORE: FtJi1-3-4, FtJi2, FtJi5, OMO 123, OMO57[1], completed by four new complexes discovered by the OMO Group Research Expedition - OGRE (PI. JRB) between 2008 and 2019: OMO 79, OMO 1/E, OMO 371N, OMO 371S. “Site complexes” correspond to areas comprising at least two distinct occurrences of either surface concentrations and/or *in situ* archaeological remains, which include a degree of stratigraphic control that allows reliable inter-occurrence correlations. Archaeological occurrences are spatially circumscribed concentrations of surface and/or *in situ* artefacts found atop or on the slopes of sedimentary hills. To date, 119 archaeological occurrences have been recorded in Member F, referred for instance to as OMO A38 for the isolated occurrences or OMO 79 – OMO A43 for the occurrences located in site complexes, abbreviated as A38, A43. Most site complexes are bracketed by minor fault systems or channel incisions which induce local stratigraphic disconformities, making it impossible for the time being to establish precise stratigraphic correlations between Member F site complexes.

Taphonomy and surface alterations of artifacts

The surface alterations or post-depositional surface modifications[2] regroup multiple wear types such as striations, abrasion, corrosion, rounding, and edge damage. What characterizes these taphonomic alterations are the randomness of their location and orientation on the surfaces and edges of an artifact. In this study we focus only on the general preservation of an artifact to evaluate the impact of post-depositional processes on the assemblages. To do so we focused firstly on the macroscopic abrasion of edges and ridges of the artifacts and secondly on the micro-fracturation of the quartz crystals and ridges, resulting in a rough surface with or without accompanying striations. This kind of alteration is sometimes called abrasion[3-5] or corrosion[6, 7] caused by “continuous pecking, wholly or almost wholly destroying the original surface”[7]. Sometimes this alteration is also referred to as “chaotic abrasion” to describe the random distribution of the taphonomic wear[4]. The process of formation of this kind of microfracturation or “breakage” of the quartz crystals is still misunderstood, as friction against abrasive materials and chemical dissolution of the original surface could be interacting before deposition and within the deposits over a long period of time. In this study we choose to qualify this type of wear as microfracturation as the origin of the alteration, mechanical and/or chemical, is unknown.

The microwear study focuses on the occurrences of over 100 artifacts. Six out of 13 occurrences were selected and only the artifacts (pebbles are not included) longer than 2 cm were observed. The macrowear and the microwear of the selected sample were directly compared in order to obtain surface alteration data on a multi-scale range. The metallographic microscope Olympus BHM equipped with differential interference contrast was used in the observation of taphonomic wear. Non-destructive analyses started with the cleaning of the artefacts using 90° alcohol and ultrasonic cleaning using neutral soap and demineralized water. Then the 200x and 400x magnification were used to observe the artifacts from their edges to the central surfaces all along the widest surface of each product type. No cortical surfaces were examined.

The proportion of each classes of abrasion at macroscopic and microscopic scales show the degree of alteration relating to different site contexts within the OMO 79 complex (Fig. S1). When macroscopic observation is useful in the determination of long water transport and intrusive pieces in the assemblages, it can be seen here that microscopic observation reveal more detailed information about the intensity of the surface alterations within a specific sedimentary deposit. The combination of both scales gives us data to discuss the impact of both depositional and post-depositional processes on the lithic assemblages.

a

b

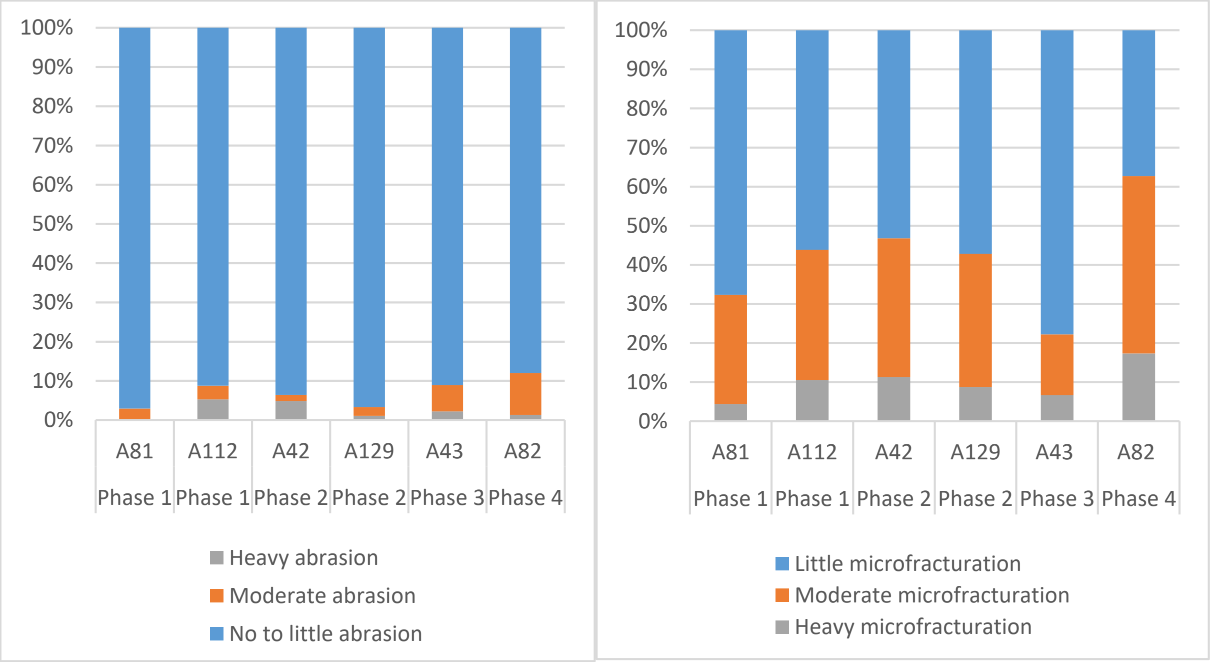

c

Phase	Occurrence	Little to no abrasion	Moderate abrasion	Heavy abrasion	Total
**1**	**A81**	66	2	0	68
**1**	**A112**	52	2	3	57
**2**	**A42**	58	1	3	62
**2**	**A129**	88	2	1	91
**3**	**A43**	41	3	1	45
**4**	**A82**	66	8	1	75
**Phase**	**Occurrence**	**Little microfracturation**	**Moderate microfracturation**	**Heavy microfracturation**	**Total**
**1**	**A81**	46	19	3	68
**1**	**A112**	32	19	6	57
**2**	**A42**	33	22	7	62
**2**	**A129**	52	31	8	91
**3**	**A43**	35	7	3	45
**4**	**A82**	28	34	13	75

Figure S1. Macro- and microwear alteration. **a.** Proportion of abraded artefacts according to each occurrence and phase of occupation. **b.** Proportion of micro-fractured artefacts according to each occurrence and phase of occupation. **c**. Detailed numbers of the selected artefacts attributed to the macroscopic (top) and microscopic (bottom) stages of alteration.

Archaeological investigations at OMO 79

Field work was carried out in 2014 and 2018 by a Franco-Ethiopian team involving Haregwin Belete, Pascal Bertran, Michel Brenet, Marion Corbé, Aline Galland, Bisrat Gebreegziabher, Misganaw Gebremichael, Brad Gravina, Tiphaine Maurin, Fikru Mekonenn, coordinated by Anne Delagnes.

OMO 79 - A43

The A43 occurrence showed abundant surface remains of the eastern side of a sedimentary hill (Fig. S2a), at the level of a natural terrace topped by carbonate concretions, nearly 10 meters below the top of the hill. A 0.50 m wide trench oriented West-East allowed to find the archaeological level *in situ* and to describe in detail the overlying sedimentary sequence. The archaeological level is part of a sequence marked by a rapid alternation of clays and silts, interpreted as overbank deposits. It is positioned at the interface between a clay deposit with carbonate concretions and the underlying silts (Fig. 2 and Fig. S2b).

The archaeological layer has been excavated over an area of 6 m² (Fig. S2c and S2d), which corresponds to most of the preserved surface of the site, estimated at 8-9 m². The relatively steep slopes of the sedimentary hill intersect the archaeological layer on its southern, northern and eastern sides (Fig. S2c). The majority of the pieces were part of a relatively compact 20-30 cm thick layer, following the general West/Southwest dip of the whole sequence. The lithic remains were associated *in situ* with small, highly altered bone fragments following the same spatial distribution (Fig. S2d and S2e). The fauna must have been originally abundant in this site, based on the many highly degraded bone fragments found during excavation, but impossible to sample. In total, only 12 small and indeterminable bone fragments could be collected at the excavation, associated with 121 *in situ* lithic remains.

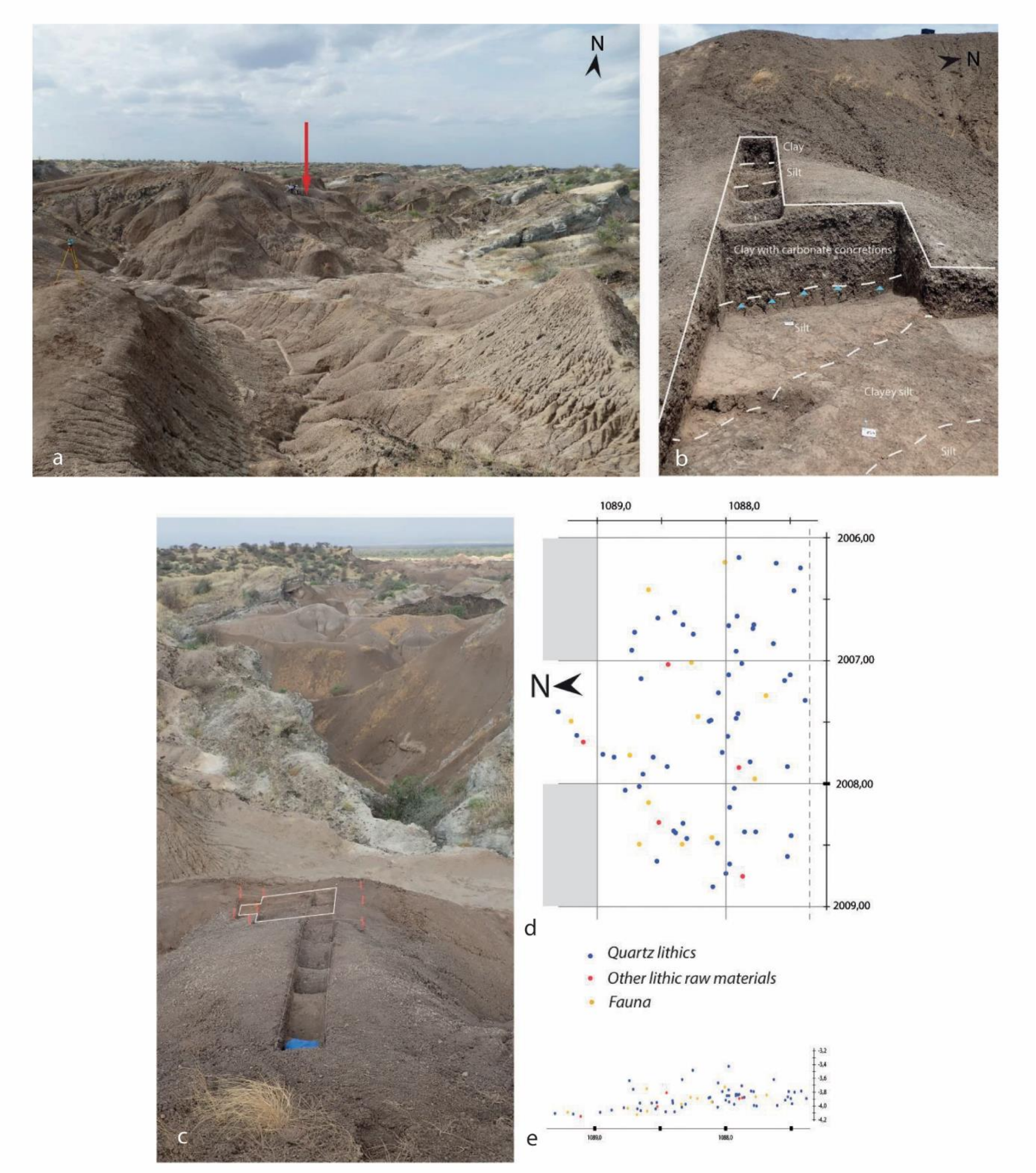

Figure S2. OMO 79 - A43 excavation. **a**. Site location (red arrow) in the OMO 79 complex, at the level of a natural terrace topped by carbonate concretions, nearly 10 meters below the top of the hill. **b.** archaeological trench and excavation; the archaeological layer, materialized by blue triangles, is part of a sequence marked by a rapid alternation of clays and silts, interpreted as overbank deposits. It is positioned at the interface between a clay deposit with carbonate concretions and the underlying silts. **c.** View of the excavation from top of the hill; a 0.50 m wide trench oriented West-East allowed to find the archaeological level *in situ* and to describe in detail the overlying sedimentary sequence. **d.** Horizontal distribution of the *in situ* archaeological remains. **e**. Vertical distribution of the *in situ* archaeological remains.

OMO 79 - A82

Located 90 meters south of A43, the A82 occurrence had yielded abundant surface remains in 2014, completed by a new collect of surface remains in 2018, on the sides of a sedimentary hill topped by a clayey level with carbonate concretions. A first trench in the zone of maximum concentration, from the top of the hill, revealed a deposit of homogeneous sandy silts more than 2 meters thick and completely sterile. The clayey deposit containing the archaeological pieces was only preserved in this area in the form of a soft surface deposit at the top of the hill (Fig. S3). A second trench carried out on the sedimentary hill (A82 South) located about ten meters south of the first (Fig. S3), revealed three quartz artifacts *in situ*, 30 cm below the top of the hill, in the upper part of a compact silty clay deposit, nearly 1.5 m thick. The sandy silt deposit capping A82 was found in A82 South directly below this clayey deposit (Fig. S3). Two *in situ* artifacts mark the position of the original archaeological layer. A 2m² excavation area was opened from the top of A82 South hill up to the height of the *in situ* artifacts in order to check the presence of a denser artifact concentration. No other archaeological remains were discovered, indicating a low density layer in this area.

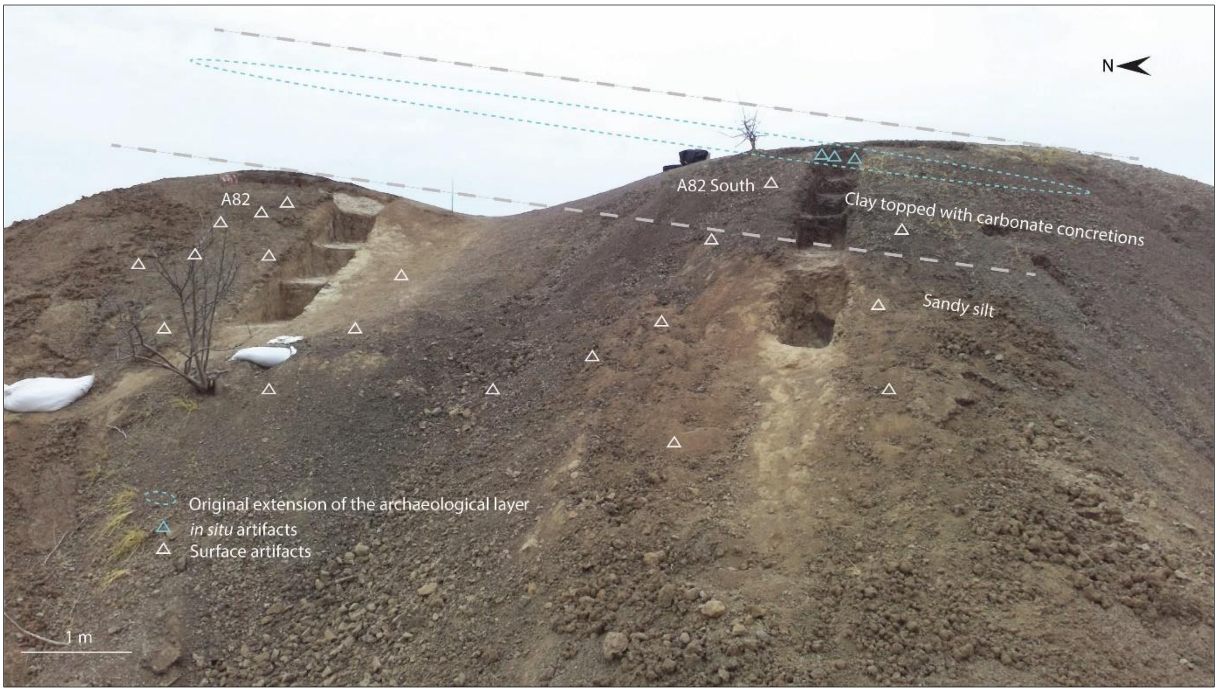

Figure S3: OMO 79 – A82. View on the western side of A82 and A82 South sedimentary hills; virtual reconstruction of the original position and extension of the archaeological deposit taking into account the Western-Southwestern dip of the deposits.

When considering the general dip of the deposits, it clearly appears that the A82 surface artifacts come from an uppermost layer which has been completely eroded in this area, while still preserved at A82 South. Characterized by less abundant surface artifacts, the A82 South area seems to correspond to an area of lower density originally situated at the margin of a site which has been largely affected by deflation. The presence of slickensides indicates synsedimentary processes that are typical of argiliturbation in contexts of alternating dry and moist phases[8,9]. Combined with deflation in the northern part of the site, argiliturbation might have significantly move the artifacts vertically, while there must not have been any significant horizontal transport. The lithic assemblage collected at A82 and A82 South nevertheless form

a homogeneous assemblage initially found in a floodplain environment. As usually observed in this type of deposit, bone remains were highly altered. Only 7 small indeterminable bone fragments were found on the surface of A82.

OMO 79 lithic assemblages

	A81	A112	A42	A129	A43	A82	Exp.
N - Lithic categories							
Flakes	26	28	20	32	23	38	437
Broken flakes	65	54	53	64	56	78	477
Flake fragments	29	21	33	38	49	87	181
Angular fragments	21	15	27	50	28	54	451
Cores	7	5	9*	5*	4	9	52
N - Raw materials							
Quartz	142	121	137	182	145	249	1598
Gneiss	5	0	4	5	1	2	-
Volcanic	0	0	1	0	4	7	-
Chert/chalcedony	1	2	0	2	7	5	-
Indet.	0	0	1	1	3	3	-
N TOTAL	**148**	**123**	**143**	**190**	**160**	**266**	**1598**

*+ 1 hammerstone

Table S1. Composition of the OMO 79 lithic assemblages. Technological composition of the archaeological assemblages compared with experimental data (Exp.) and raw material frequencies (see https://doi.org/10.34847/nkl.3e292r29 for the experimental dataset): occurrences A81, A42, A43, A82, A112, A129, including all lithics > 1 cm, and excluding the unmodified clasts.

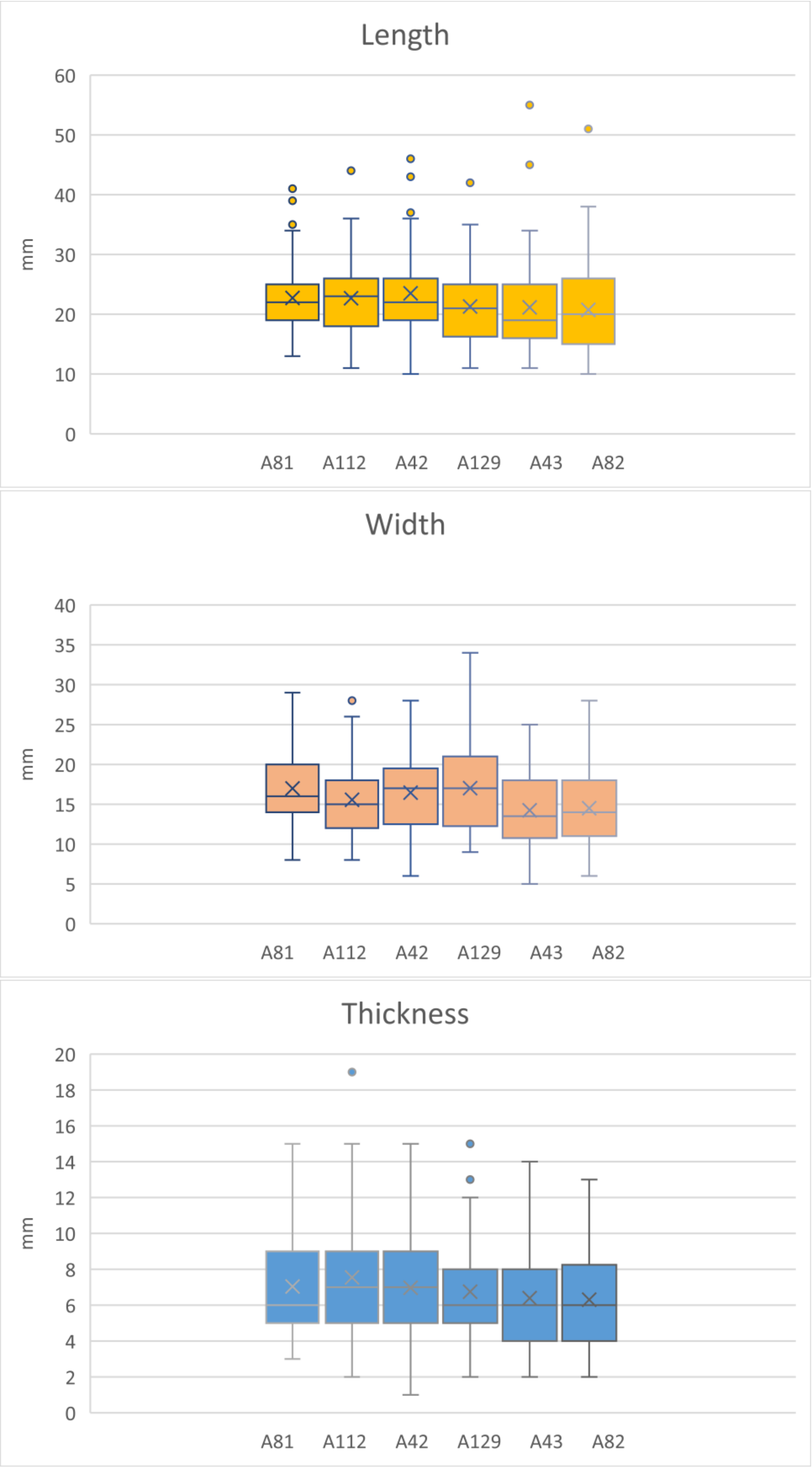

Figure S4. Omo 79 - Boxplots for the dimensions of the flakes and broken flakes. Maximum length, maximum width perpendicular to length and maximum thickness of all flakes and broken flakes > 1 cm. Source data, mean values and standard deviations are available on Nakala repository at https://doi.org/10.34847/nkl.052d9a30.

OMO 79 faunal assemblages

Faunal material from phase 1 is particularly abundant in the south of the site complex at A112. In this alluvial sand deposit, a clear association between bones and lithic artifacts cannot be established. Bone remains from phase 2 are particularly abundant north of the site complex, in A42 where abraded bones account for almost 10% of the total faunal assemblage[10]. No evidence of human modifications were recorded on any of the faunal remains despite a relatively large proportion of bone fragments with unaltered surfaces[10]. At A43 (phase 3), numerous heavily altered and 12 better-preserved bones were found in primary spatial association with the quartz artifacts, suggesting the original faunal assemblage was substantially larger. At A82 (phase 4), a small number of tiny heavily abraded and indeterminable bone fragments were collected from the surface. Bone remains are thus poorly informative in this context due to the depositional dynamics evidenced for phases 1 and 2 which prevents any direct correlation with the archaeological remains, and to their bad preservation for phases 3 and 4 assemblages.

References

1. Howell, F. C., Haesaerts, P. & de Heinzelin, J. Depositional environments, archeological occurrences and hominids from Member F and E of the Shungura Formation (Omo Basin, Ethiopia). *J Hum Evol* **16**, 665-700 (1987).
2. Holmes, D. L. Problems encountered in a high-power microwear study of some Egyptian predynastic lithic artefacts. In The Human Uses of Flint and Chert*,* G. Sieveking, M. Newcomer, Eds. (Cambridge University Press, 1987), pp. 91-96.
3. Knutsson, K. *Patterns of tool use: scanning electron microscopy of experimental quartz tools* (Societas Archaeologica Upsaliensis Uppsala, 1988).
4. Venditti, F., Tirillò, J. & Garcea, E. A. Identification and evaluation of post-depositional mechanical traces on quartz assemblages: An experimental investigation. *Quat. Int.* **424**, 143-153 (2016).
5. Sussman, C. E. *Functional Studies of Experimental Quartz Artefacts Using Microscopic Analysis of Use-Wear and Polish Formation (Lithics)*. (University of California, Berkeley), 253 p. (1986)
6. Gibaja, J. F., Clemente, I. & Carvalho, A. F. The use of quartzite tools in the early Neolithic in Portugal: Examples from the limestone massif of Estremadura. in *Recent functional studies on non flint stone tools: methodological improvements and archaeological inferences - Proceedings of the Workshop (Lisboa)* (2009)
7. Clemente, I. & Gibaja, J. Formation of use-wear traces in non-flint rocks: the case of quartzite and rhyolite. Differences and similarities. In Non flint Raw Material Use in Prehistory: Old Prejudices and New Directions, 93-98 (2009).
8. Delagnes, A. *et al.*, Interpreting pachyderm single carcass sites in the African Lower and Early Middle Pleistocene record: a multidisciplinary approach on the site of Nadung'a 4. *Journal of Anthropological Archaeology* **25**, 448-465 (2006).
9. Eswaran, H., Beinroth, F. H., Reich, P. F. & Quandt, L. A. Eds., *Vertisols : their properties, classification, distribution and management.* (Soil Survey Division. USDA Natural Resource Conservation Service, Washington, 1999).
10. Maurin, T., Bertran, P., Delagnes, A. & Boisserie, J.-R. Early hominin landscape use in the Lower Omo Valley, Ethiopia: Insights from the taphonomical analysis of Oldowan occurrences in the Shungura Formation (Member F). *J Hum Evol* **111**, 33-53 (2017).
